# Availability and utilization of the WHO recommended priority lifesaving medicines for under five-year old children in public health facilities in Uganda: a cross-sectional survey

**DOI:** 10.1186/s40545-015-0038-2

**Published:** 2015-05-11

**Authors:** Xavier Nsabagasani, Jasper Ogwal-Okeng, Anthony Mbonye, Freddie Ssengooba, Simon Muhumuza, Ebba Holme Hansen

**Affiliations:** Child Health and Development Center, College of Health Sciences, Makerere University, P.O. Box 6717, Kampala, Uganda; Department of Pharmacology and Therapeutics, Gulu University, Gulu Municipality, Uganda; Ministry of Health Uganda and Department of Health, Uganda Christian University, Kampala, Uganda; School of Public Health, College of Health Sciences, Makerere University, Kampala, Uganda; Section for Social and Clinical Pharmacy, Department of Pharmacy, Faculty of Health and Medical Sciences University of Copenhagen, Copenhagen, Denmark

**Keywords:** Lifesaving priority medicines, Children under five, Public health facilities, Uganda

## Abstract

**Objectives:**

To explore the availability and utilization of the World Health Organization (WHO) recommended priority life-saving medicines for children under five in public health facilities in Uganda.

**Methods:**

We conducted a cross sectional survey in 32 lower level public facilities in Jinja district of Uganda. A proportionate number of facilities were randomly selected in each stratum following a hierarchy of Health Centers (HC) defined according to the level of care they provide: 17 HC IIs, 10 HC IIIs and 5 HC IVs. In the facilities, we verified the availability of the WHO recommended priority medicines for diarrhea, sepsis, pneumonia and malaria. 81 health workers from the facilities reported what they prescribed for children with the above diseases.

**Results:**

Oral rehydration salt (ORS) and zinc sulphate dispersible tablets for diarrhea were available in all HC IIs and IIIs and in only 60% of HC IVs. Procaine benzyl penicillin injection powder for treatment of sepsis was available in the majority of all HCs with: 100% of HC of IVs, 83% of HC IIIs and 82% of HC IIs. Medicines for pneumonia were limited across all the HCs with: Amoxicillin dispersible tablets in only 30% of the HC IIs and 40% of the HC IVs. The most uncommon were child-friendly priority medicines for malaria with: Artesunate injection in only 6% of HC IIs, 14% of HC IIIs and 20% of HC IVs; Artemether lumefantrine dispersible tablets and rectal artesunate were missing in all the 32 HCs. Less than a third of the health workers reported prescribing zinc sulphate and ORS for diarrhea, 86% reported procaine benzyl penicillin injection powder for sepsis, and 57% reported amoxicillin dispersible tablets for pneumonia. None reported prescribing Artemether lumefantrine dispersible tablets and rectal artesunate for malaria.

**Conclusions:**

There is low availability and utilization of life-saving priority medicines for pneumonia and malaria in public health facilities in Uganda. However, the priority medicines for diarrhea and sepsis are available and highly prescribed by the health workers.

## Introduction

Globally, an estimated 8.1 million children under five die every year due to conditions that could be prevented or treated with evidence-based medicines [1]. Many parents and caretakers cannot afford treatment and consultations needed by their children. Even where some parent can afford the medicines, these are often not available [2]. A study by Robertson and colleagues showed poor availability and accessibility of children’s medicines and in order to achieve substantial progress towards Millennium Development Goals (MDGs), a major effort to improve access to medicines for children will be required [3]. In order to achieve the desired therapeutic outcomes for children, access to age appropriate and well tolerated drug formulations is essential [4,5]. Hence the need for countries to have essential medicines list for children [6]. However, access to appropriate medicines for children is a major challenge [7]. Studies have shown that child appropriate medicines do not reach the children who need them [5]. In 2007, the World Health Assembly (WHA) resolution 60.20 urged countries to promote access to medicines for children [8]. In the same year, the World Health Organization (WHO) and partners launched the Make Medicines Child Size Campaign to increase children’s access to appropriate dosage formulations [9]. The need to access appropriate medicines for children is recognized as an essential step in achieving the Millennium Development Goals 4 and 6 [3,10].

The Make Medicines Child Size Campaign (MMCS) highlighted the challenges of relying on oral liquid dosage forms for children below 8 years, on the basis that this age group has difficulties in swallowing capsules and tablets. Syrups are inappropriate for low income countries because of high cost, short shelf life and the requirement for appropriate storage and logistical management systems. Thus, alternative formulations which are palatable and easy to administer to children were considered [10]. Hence, in 2008, flexible oral solid dosage formulations were introduced to address the shortcomings of syrups [11]. Flexible oral solid dosage formulation can be orally administered to children in the form of disintegrating mini-tablets, dispersible tablets or effervescent tablets [12].

The significance of essential medicines has been emphasized by some scholars. Funding for essential medicines is essential to ensure access to the medicines for the patients who need the medicines [13]. Holloway and Henry concluded that the WHO essential medicines policies are associated with improved Quality Use of Medicines (QUM), particularly in low-income countries [14]. Increasing children’s access to essential medicines requires developing an essential medicines list for children and including these medicines in national purchasing lists [15]. In 2011, the WHO, UNICEF and UNFPA developed a list of priority medicines for mothers and children [1]. A year later, the list was revised and renamed priority life-saving medicines for women and children, following the 18th Expert Committee Meeting on Selection and Use of Medicines [16]. Among the key diseases targeted in the recommended priority list for children under five were pneumonia, neonatal sepsis, diarrhea and malaria. The key dosage formulations for treating these conditions that were emphasized included scored, dispersible tablets and injectables. A study conducted in 129 public health facilities in India showed that availability of essential medicines for children in public health facilities is not sufficient and needed improvement [17]. A study conducted in 40 public hospitals, 40 private hospitals and 8 pharmacies in Sri Lanka showed that the key essential medicines for children were less available in public hospitals than in private and pharmacies [18].

Despite the introduction of priority medicines for children under five at the global level, studies conducted in India, Chad, Kenya and some parts of Uganda showed limited availability of the medicines in public health facilities [19-21]. Little is known about the availability and utilization of the priority medicines for treating under five-year-old children in public health facilities in many parts of Uganda. This study explored the availability and utilization of the WHO recommended life-saving priority medicines for under five-year-olds in lower level public health facilities in the Jinja district of Uganda.

## Methods

### Study design

This was a cross-sectional survey conducted between June and August 2012 in Jinja District, south-eastern Uganda. The survey employed quantitative methods of data collection.

### Study setting

The study was part of a 5-year multidisciplinary project (the ChildMed project) that aimed at improving medicine use and management for children in Uganda (childmed.ku.dk). This study was conducted during the third year of the project.

The district has a population of more than 501,000 people, 79.1% of whom live in rural areas. More than half (56%) of the population in the district is below 18 years of age [22]. Administratively, the district is subdivided into seven rural sub-counties, one town council and three divisions in the municipality.

The health care system in the district, like the rest of the country, is organized under a hierarchy of health facilities; HC I, II, III and IV. The higher level facilities supervise the lower level units. HC IV facilities are located at county (constituency) level with an estimated catchment population of about 100,000. HCs III are located at sub-county level and serve a catchment population of about 20,000, while HCs II are located at parish level and have a catchment population of about 5,000. HCs I are located at village (Local Council I) level and are composed of community volunteers referred to as village health teams (VHTs). These constitute the lowest structure in the district health care system. VHTs’ key responsibilities include identifying the community’s health needs, mobilizing communities for utilization of the resources, mobilizing communities for health interventions and promoting health seeking behaviors [23].

Jinja district has a total of 69 health facilities, 49 of which are public, seventeen are run by NGOs and three are institutional (army, police and prisons). There is 100% health facility coverage per parish with the majority of the population living within a 5 km radius of a health facility. The district has health worker coverage of 73.2% in both public and private health facilities (MOH, 2010/2011). The major causes of morbidity and mortality in the area include malaria, acute respiratory infections, HIV/AIDS, diarrhoeal diseases and complications of pregnancy and trauma.

### Sample size

Two thirds of the public lower level facilities in Jinja district (32) were studied: 17 HC IIs, 10 HC IIIs and 5 HC IVs. A total of 81 health workers from the same facilities were purposively selected to participate in the interviews. Table 1 provides details of the sample size for health workers selected at each level of health facility.

**Table 1:** **Sample of the respondents according to the level of the facility and qualifications**

	**Qualifications of the respondent**						
**Health facility level**	**Nursing assistant**	**Registered midwife**	**Enrolled nurse**	**Enrolled midwife**	**Registered nurse**	**Public health nurse**	**Total number in the level of health facility**
HC II	16	3	3	0	9	0	31
HC III	10	5	6	4	3	1	29
HC IV	8	6	3	3	1	0	21
**Total**	**34**	**14**	**12**	**7**	**13**	**1**	**81**

### Sampling procedure

A stratified one-stage cluster design was employed to select the facilities. All the lower level public health facilities were stratified according to the level of care they provide. A proportionate number of health facilities to participate in the study from each stratum were determined by probability proportional to size of the health facility population in the stratum. Systematic sampling, using a list of facilities in each stratum as the sampling frame, was employed. The sampling interval was obtained by dividing the total number of facilities with the number of facilities to be studied in each stratum (N/n). After obtaining a random start from a table of random numbers, the interval was followed until the required number of facilities in each stratum was obtained.

In each of the selected health facilities, health workers who were treating the children at the time of the visit and were willing to participate in the study were purposively selected. Two to three health workers were selected from each facility.

### Data collection

There were two key components of the study; the first component established the availability of priority medicines in the drug stores/pharmacies in the selected facilities covering medicines for pneumonia, diarrhea, malaria and neonatal sepsis. Availability of the priority medicines in their WHO recommended strengths and dosage formulations were assessed through physical verification of the medicines in the stores/pharmacies of the health facilities using a checklist.

The second component included face to face interviews with the selected health workers using a structured questionnaire. Interviews were conducted by trained research assistants.

### Study variables

The study focused on the WHO recommended priority life-saving medicines for children under-five for (1) pneumonia: amoxicillin dispersible scored tablets, ampicillin powder for injection, ceftriaxone powder for injection, and Gentamycin injection (2) diarrhea: zinc sulphate dispersible tablets and Oral Rehydration Salts Sachets (3) malaria: ACTs, rectal artesunate and artesunate injectable and (3) neonatal sepsis: Ampicillin injectable and Procaine benzyl penicillin powder for injection. In addition, the health workers were asked to mention the names and dosage forms of the medicines that they were using to treat pneumonia, sepsis, diarrhea and malaria among children under five.

### Data management and statistical analysis

Data entry and validation were performed in Epi-Info software (version 7.1.2; Centers for Disease Control and Prevention, Atlanta (Georgia), US) and exported to the Statistical Package for Social Scientists (SPSS 12.0) for descriptive analysis. Analysis was done at univariate level to generate frequencies, means and proportions. Availability was calculated using the number of the WHO recommended priority life-saving medicines for diarrhea, pneumonia, malaria and sepsis among children under five as a percentage of the total number of surveyed facilities. Similarly, utilization was calculated using the reported medicines prescribed as a percentage of the total number of health workers interviewed.

### Quality control

Experienced research assistants were recruited and trained in data collection methods. The questionnaires were pre-tested in one of the health facilities excluded in the study for purposes of clarity, validation, suitability, and logical flow of the questions. Interviews with the health workers were held in a private room within the health facility. Medicines were checked with the help of the dispenser by physically verifying the medicines in the shelves. Data were checked for completeness and accuracy before leaving the facility and as such, no data were missing. The first author closely supervised the research assistants during data collection.

### Ethical considerations

The study was approved by the School of Medicine Research and Ethics Committee of the College of Health Sciences, Makerere University, and was cleared by the Uganda National Council of Science and Technology. Further clearance was obtained from the District Health Officer, Jinja district. Permission to conduct the study was obtained from the officials in-charge of the health facilities. Written informed consent was obtained from the health workers who participated in the interviews. They were also informed that their participation in the study was voluntary and that they could withdraw any time they wished.

## Results

### Availability of the WHO recommended life-saving medicines for under five-year-olds

Overall, considerable variations in availability of the WHO recommended life-saving medicines for children under five were noted across the health facility levels.

### Priority medicines for diarrhea and sepsis

Oral rehydration salt (ORS) and zinc sulphate dispersible tablets for treatment of diarrhea were available in all the HC IIs and IIIs but present in only 60% of the HC IVs. Procaine benzyl penicillin injection powder for treatment of sepsis was available in the majority all HCs; 100% of the HC IVs, 83% HC IIIs and 82% of the HC IIs (Table 2).

**Table 2:** **Availability and utilization of life saving priority by health workers to treat children under five**

**Illness**	**WHO recommended medicine and dosage form**	**Dosage form included in EMHSLU (1 = Yes 2 = No) (classification as vital/non-vital for the level)**	**Availability [N = 32 (%)]**			**Utilization by health workers (N = 81) %**
			**HC II (N = 17)**	**HC III (N = 10)**	**HC IV (N = 5)**	
**Pneumonia**	Amoxicillin Dispersible, scored tablets 250 mg; 500 mg	**2** (non-vital for all HC levels)	60 (30)	0 (00)	02 (40)	46 (57)
	Ampicillin Powder for injection 500 mg; 1 g	**1** (non-vital for HC II, vital for HC III & IV)	00 (00)	04 (40)	02 (40)	20 (24)
	Ceftriaxone Powder for injection 500 mg; 1 g	**1** (non-vital for HC II & III, vital for HC IV)	00 (00)	00 (0)	03 (60)	4 (5)
	Gentamycin Injection 40 mg/ml; 20 mg/ml	**1** (non-vital for HC II, vital for HC III & IV)	04 (20)	06 (60)	02 (40)	30 (37)
**Diarrhoea**	Oral Rehydration Salt (ORS): Sachets of 200 ml; 500 ml; 1000mls	**1** (vital for all HC levels)	20(100)	10 (100)	03 (60)	15 (19)
	Zinc sulphate 20 mg: Dispersible tablet	**1** (vital for all HC levels)	16 (94)	10 (100)	03 (60)	20 (25)
**Malaria**	Artemesinin combination therapy (ACT): Dispersible tablets	**2** (non-vital for all HC levels)	00 (0)	00 (00)	00 (00)	0 (00)
	Artesunate Injection 50–200 mg	**1** (vital for all HC levels)	01 (10)	01 (14.3)	01 (20)	0 (00)
	Artesunate Rectal 50–200 mg	**1** (vital for all HC levels)	0 (00)	0 (00)	0 (00)	0 (00)
**Neonatal sepsis**	Procaine benzyl penicillin; injection 1 g	**1** (non-vital for HC II, vital for HC III & IV)	14 (82)	06 (86)	05 (100)	70 (86)

### Priority medicines for pneumonia and malaria

Medicines for the treatment of pneumonia medicines were limited across the HCs with Gentamycin injection present in 20% of the HC IIs, 60% of the HC IIIs, and in 40% of HC IVs. Ampicillin injection powder was absent in all the HC IIs but present in only 40% of the HC IIIs and HC IVs Similarly, Amoxicillin dispersible tablets were present in only 30% of the HC IIs and 40% of the HC IVs. Artesunate injection for malaria was available in only 6% of the HC IIs, 14% of the HC IIIs and 20% of the HC IVs; Artemether lumefantrine dispersible tablets and rectal artesunate were not available in all the 32 Health facilities that were surveyed (Table 2).

### Utilization of the priority life-saving priority medicines for children under five

In terms of what is prescribed for children, less than a third of the health workers reported zinc sulphate and ORS for diarrhea, 86% reported procaine benzyl penicillin injection powder for sepsis, and 57% reported amoxicillin dispersible tablets for treating pneumonia. None of the health workers reported Artemether lumefantrine dispersible tablets and rectal artesunate for malaria (Table 2).

## Discussion

This study found that there is low availability and utilization of the WHO recommended lifesaving priority medicines for pneumonia and malaria in most public health facilities. Nonetheless, the medicines for diarrhea and sepsis were highly available. However, utilization of the medicines for diarrhea was very low.

In low-income countries, pneumonia and malaria are the leading cause of morbidity and mortality among children under five years of age [24]. Thus, increasing access to treatment for pneumonia and malaria in this age-group is critical. In this study, dispersible Artemether lumefantrine and rectal and injectable artesunate, the WHO recommended life-saving priority medicines for malaria, were not available in the health facilities surveyed. This finding is in line with the Ugandan policy provision in which the dispersible tablets were not included in the Essential Medicine and Health Supplies List for Uganda (EMHSLU) 2012. Artemether-lumefantrine, an ACT, is included in the EMHSLU as a vital medicine at the HC I and above [25] and is the recommended first line treatment for malaria [26,27]. However, the WHO recommended medicine formulation (dispersible tablet of Artemether-lumefantrine) is not available at all levels of care and as such, the health workers do not prescribe it for children. Rectal and injectable artesunate which are also classified as vital in the EMHSLU 2012 at the lowest levels of health facilities share the same fate. This is despite the fact that Artesunate is an important pre-referral medicine for severe malaria at the lowest health facility levels. Artesunate is easy to administer to children in both the injectable and suppository formulations [26,27].

Uganda has undertaken medicine policy reforms that have influenced medicine availability. Between 2010 and 2012, both the Uganda Clinical Guidelines (UCG) and the Essential Medicines List (EML) were revised to accommodate the new evidence medicines and to get rid of some dosage forms such as syrups. The 2012 Essential Medicines and Health Supplies List of Uganda (EMHSLU) introduced the Vital, Essential and Necessary (VEN) classification of medicines [25,26]. The “Vital” (V) medicines are used to manage life-threatening diseases, “Essential” (E) medicines are effective in management of less severe, but nevertheless, widespread illnesses and “Necessary” (N) medicines are used to treat diseases with less impact on the population or items with a high cost for marginal therapeutic benefit [25]. The overall purpose for introducing the VEN classification was to enable health facility and ministry of health procurement officials to prioritize the medicines to procure. The VEN classification does not emphasize dosage formulations for children. This explains why some of the vital medicines such as Artemether Lumefantrine were not available in the dosage formulations (dispersible tablets) recommended by WHO.

It has been argued that prescribing drug formulations which are easy to administer may foster compliance and improve therapeutic outcomes in infants and young children. The reverse is true where such formulations are unavailable [28]. Hence, gaps in the provisions for some of the medicines in the EMHSLU 2012 corroborate with their absence at the lower level facilities further demonstrating policies provisions influence availability and sometimes utilization.

This study showed a positive direction towards the use of evidence-based and child appropriate formulations to treat pneumonia. However, this was still very low. The WHO recommends a replacement of cotrimoxazole as the first line treatment for pneumonia among under five-year-old children with amoxicillin dispersible tablets [29]. This is because cotrimoxazole is only effective against non-severe pneumonia [30] and less effective against severe pneumonia compared to amoxicillin [31], especially in settings where HIV prevalence is high [32,33]. In the study area, amoxicillin dispersible tablets were present in only 30% and 40% of the HC IIs and IVs surveyed, respectively, but were conspicuously absent in all the HC IIIs. According to UNICEF, more than 1.5 million lives of children could be saved if amoxicillin was available in the health facilities [34].

Zinc sulphate dispersible tablets and ORS are provided for in the EMHSLU and the Uganda Clinical Guidelines [25-27], classified as Vital for all the levels of health facilities [25]and were also available in most of the health facilities surveyed. However, it is surprising that more than two thirds of the health workers did not mention their use in the treatment of diarrhoeal diseases among under-five children. The WHO in collaboration with the United Nations Children Education Fund (UNICEF) and the United Nations Population Fund (UNFPA) recommended the use of ORS sachets and zinc sulphate scored dispersible table or equivalent flexible oral solid dosage form for the treatment of diarrhea in children [1,16]. In addition, it has been argued that zinc supplementation reduces the duration and severity of acute and persistent diarrhea and prevention of pneumonia and other diseases in children [35,36]. Thus, the limited use of zinc sulphate among children with diarrhea remains a critical gap that needs to be addressed all levels.

### Study limitations

This study did not explore the factors affecting the availability and utilization of the priority medicines for children in public health facilities in Uganda. Undertaking a more in-depth study to explore the underlying factors is needed. Secondly, majority of the health workers interviewed were nursing assistants because this cadre of staff was the most available at the health facilities during the study visit. It is possible that the views and practices of the senior staff in relation to priority medicines for children explored in this study were not adequately represented.

## Conclusions

This study reveals that despite the WHO emphasis, availability and utilization of the life-saving priority medicines for treating pneumonia, and malaria among the under five-year-old children were limited across the different levels of the public health facilities. Utilization of the highly available priority medicines for diarrhea and sepsis was low. There is need to integrate the WHO recommended life-saving priority medicines into the health unit logistic and essential drug management systems to increase their availability and utilization. Training of the health workers in the use of the priority medicines will be an essential step in increasing their utilization.
